# 16S metagenomics dataset of *Zea mays* and *Triticum aestivum* rhizosphere from Kallar Syedan Punjab, Pakistan

**DOI:** 10.1016/j.dib.2022.108057

**Published:** 2022-03-13

**Authors:** Sadia Latif, Rizwana Kousar, Anum Fatima, Hina Fatimah, Saba Farooq, Naeem Khan, Tayyaba Andleeb, Tariq Shah

**Affiliations:** a Departmentof Biology and Environmental Science, Allama Iqbal Open University, Islamabad, Pakistan; b Department of Agronomy, IFAS, University of Florida, Gainesville, USA; c Agroécologie, Agrosup Dijon, CNRS, INRAE, Université de Bourgogne, Bourgogne Franche-Comté, France

**Keywords:** *Triticum aestivum* L., *Zea mays* L., Rhizosphere, 16S rRNA, IonS5^tm^XL sequencing, Bacterial community

## Abstract

Plant microbiome referred to as plant second genome, plays pivotal role in determination of vigor and productivity of plant. Current high-throughput sequence technologies provide remarkable insight into microbial diversity and host microbe interaction. The obtained dataset aimed to reveal the core bacterial community residing the rhizosphere of two leading cereal crops *Zea mays* and *Triticum aestivum* grown in different seasons at the same geographical area. The rhizosphere bacterial communities were explored via amplicon sequencing of V3–V4 region of 16S rRNA region using IonS5™XL sequencing platform. The classified tags for 16S rRNA from both the samples were clustered into 1502 Microbial operational taxonomic units (OTUs) at 97% similarity with 1340 OTUs in *Zea mays* and 1337 OTUs in *Triticum aestivum*. Ten bacterial phyla predominant in the rhizosphere were *Proteobacteria, Actinobacteria, Firmicutes, Acidobacteria, Bacteroidetes, Chloroflexi, Gemmatimonadetes, Verrucomicrobia, Nitrospirae* and *Thermomicrobia*. These bacterial phyla accounted for 98% and 98.9% of the OTUs in *Zea mays* and *Triticum aestivum,* respectively. Statistical analysis depicted the presence of slight variations in the relative abundance of bacterial groups residing the rhizosphere of *Zea mays* and *Triticum aestivum*. The community data produced in the present work can be used for meta-analysis studies to understand rhizosphere bacterial community of two major cereal crops. Furthermore, bacterial composition and diversity data is prerequisite for rhizosphere engineering to enhance cereal production to cope with upcoming global challenges of climate change and population growth.

## Specifications Table

**Table:** 

Subject	Biological Sciences
Specific subject area	16S Metagenomics of Maize and Wheat Rhizosphere
Type of data	Amplicon sequencing data of V3-V4 region of 16S rRNA region
How the data were acquired	IonS5™XL sequencing platform, Quantitative Insights Into Microbial Ecology software (Qiime Version 1.7.0), UCHIME algorithm, Uparse software (Version 7.0.1001), Mothur software (SILIVA Database), Phyloseq, microbiome, knitr and dplyr packages in R-software (version 3.6.3) and XLSTAT-2021
Data format	Raw, filtered and analyzed
Description of data collection	DNA isolation from the rhizospheric soils of *Zea mays* and *Triticum aestivum*, amplification and sequencing of V3-V4 region of 16S rRNA and raw data processing using Qiime Version 1.7.0, effective read assigned to OTUs by employing Uparse software, annotation of OTUs using Mothur software, alpha diversity analysis using phyloseq, microbiome, knitr and dplyr packages and comparison between the rhizosphere bacterial community of *Zea mays* and *Triticum aestivum*.
Data source location	**Geographical oridinates:** 33.42 N° and 73.37 E° **City/Province:** Kallar Syedan, Punjab **Country:** Pakistan
Data accessibility	The clean sequences obtained were submitted as Sequence Read Archive in the National Center for Biotechnology Information under Project ID: PRJNA544498 as biosample SAMN11841210: Zea_mays_KS and SAMN11841209: Triticum_aestivum_KS with Accession Number: SRX5934262 and SRX5934263. The OTUs data for 16S rRNA has been deposited at DDBJ/EMBL/GenBank under Accession Number: KDQE00000000 (KDQE01000001-KDQE01001714). Direct URL to data: https://www.ncbi.nlm.nih.gov/sra/SRX5934262; https://www.ncbi.nlm.nih.gov/sra/SRX5934263; https://www.ncbi.nlm.nih.gov/nuccore/KDQE00000000.1/

## Value of the Data

• The myriad of rhizosphere bacteria regulates growth and development of plant. Unraveling the rhizosphere bacterial community using metagenomics provides a deeper understanding of rhizosphere setting.
• The dataset unraveled taxonomic composition of bacterial community from the rhizosphere of two leading cereal crops *Zea mays* and *Triticum aestivum* grown in different seasons at the same geographical area.
• The crop researchers working on rhizosphere microbiome can use the dataset to interlink taxonomic information with potential functional description. Functional networking of rhizosphere microbiome will help researchers in improving crop yield under adverse conditions to meet future food demand.
• The crop scientists can use dataset to perform meta-analysis studies to correlate present findings with rhizosphere microbiome research outcomes available in literature to answer the questions regarding cereal crops growth, yield and adaptability.
• The dataset produce is a baseline for further studies on plant type and seasonal shift role in modeling bacterial composition and diversity of rhizosphere.

## 1 Data Description

### 1.1 Bulk soil analysis

The bulk soil samples collected from agriculture fields of Kallar Syedan, Pakistan were subjected to soil analysis. The value for electrical conductivity, pH, organic matter, available phosphorus, potassium and saturation percentage varied between 0.72–0.79 dsm^−1^, 7.67–7.77, 0.71–1.21, 6.9–7.1 mg kg^−1^, 100–120 mg kg^−1^ and 34–36, respectively. The soil was characterized slightly alkaline and loam in texture (Table 1).

**Table 1:** Analysis of the bulk soil collected from Kallar Syedan.

Soil Depth (Inch)	Electrical Conductivity (dsm^−1^)	pH	Organic Matter (%)	Phosphorous (mg kg^−1^)	Potassium (mg kg^−1^)	Saturation (%)	Texture
1–6	0.72	7.77	1.21	7.1	100	34	Loam
7–12	0.79	7.67	0.71	6.9	120	36	Loam

### 1.2 16S rRNA metagenomics of rhizosphere

#### 1.2.1 Tags statistics

The amplicon sequencing of V3-V4 region of 16S rRNA was used to explore the core bacterial community structure residing the rhizosphere of *Zea mays* and *Triticum aestivum*. A total of 70,208 high-quality reads (total tags) were obtained from rhizosphere of *Zea mays*. Of the high quality reads, 33,971 were taxon tags (classified tags) and 36,237 were unique tags (unique reads). Whereas the total tags obtained from the rhizosphere of *Triticum aestivum* were 73,174. Of the total effective tags, 32,783 were taxon tags and 40,391 were unique tags.

#### 1.2.2 Classified tags

Of the total 70,208 high quality bacterial tags obtained from the rhizosphere of *Zea mays*, 33,971, 33,904, 33,357, 30,137, 27,070, 19,230 and 4169 were assigned to kingdom, phylum, class, order, family, genus and species rank, respectively. Whereas of the total 73,174 bacterial tags obtained from rhizospheric soil of *Triticum aestivum*, 32,738, 32,493, 30,507, 28,086, 21,131 and 3521 were assigned to phylum, class, order, family, genus and species, respectively.

#### 1.2.3 Bacterial operational taxonomic units

**Fig. 1:**
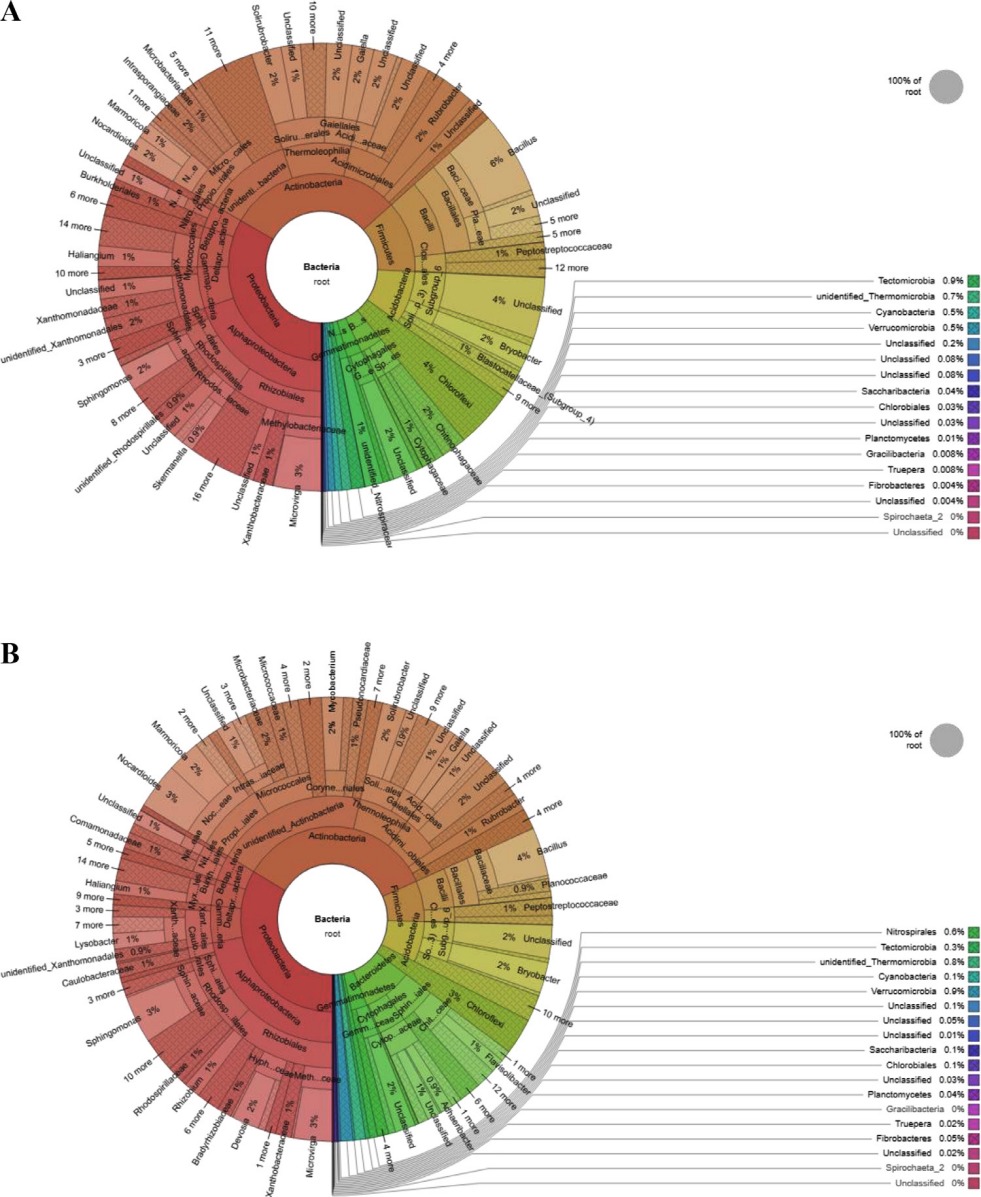
(A) The bacterial community structure of *Zea mays* rhizosphere. Krona depicted abundance and hierarchy simultaneously. Circles from inside to outside stand for different taxonomic ranks and the area of sector means respective proportion of different OTUs. (B) The bacterial community structure of *Triticum aestivum* rhizosphere. Krona displayed abundance and hierarchy simultaneously. Circles from inside to outside stand for different taxonomic ranks and the area of sector means respective proportion of different OTUs.

The bacterial tags from both the samples were clustered into 1502 Microbial operational taxonomic units (OTUs) at 97% similarity with 1340 OTUs in *Zea mays* and 1337 OTUs in *Triticum aestivum*. The OTUs obtained from *Zea mays* were classified into 22 phyla, 66 classes, 91 orders, 188 families and 284 genera. The OTUs obtained from *Triticum aestivum* were classified into 21 phyla, 65 classes, 88 orders, 184 families and 279 genera. The OTUs clustering elucidated the presence of 1175 OTUs in the rhizosphere soils of both the crops, whereas 165 and 162 OTUs were unique in the rhizosphere of *Zea mays* and *Triticum aestivum*, respectively. The OTUs found in both the rhizosphere belonged to 21 phyla, 59 classes, 80 orders, 169 families and 254 genera (Fig. 1A and B, Supplementary Table S1).

#### 1.2.4 Dominant taxonomic groups forming the core bacterial community structure

*Dominant bacterial phyla.*

**Fig. 2:**
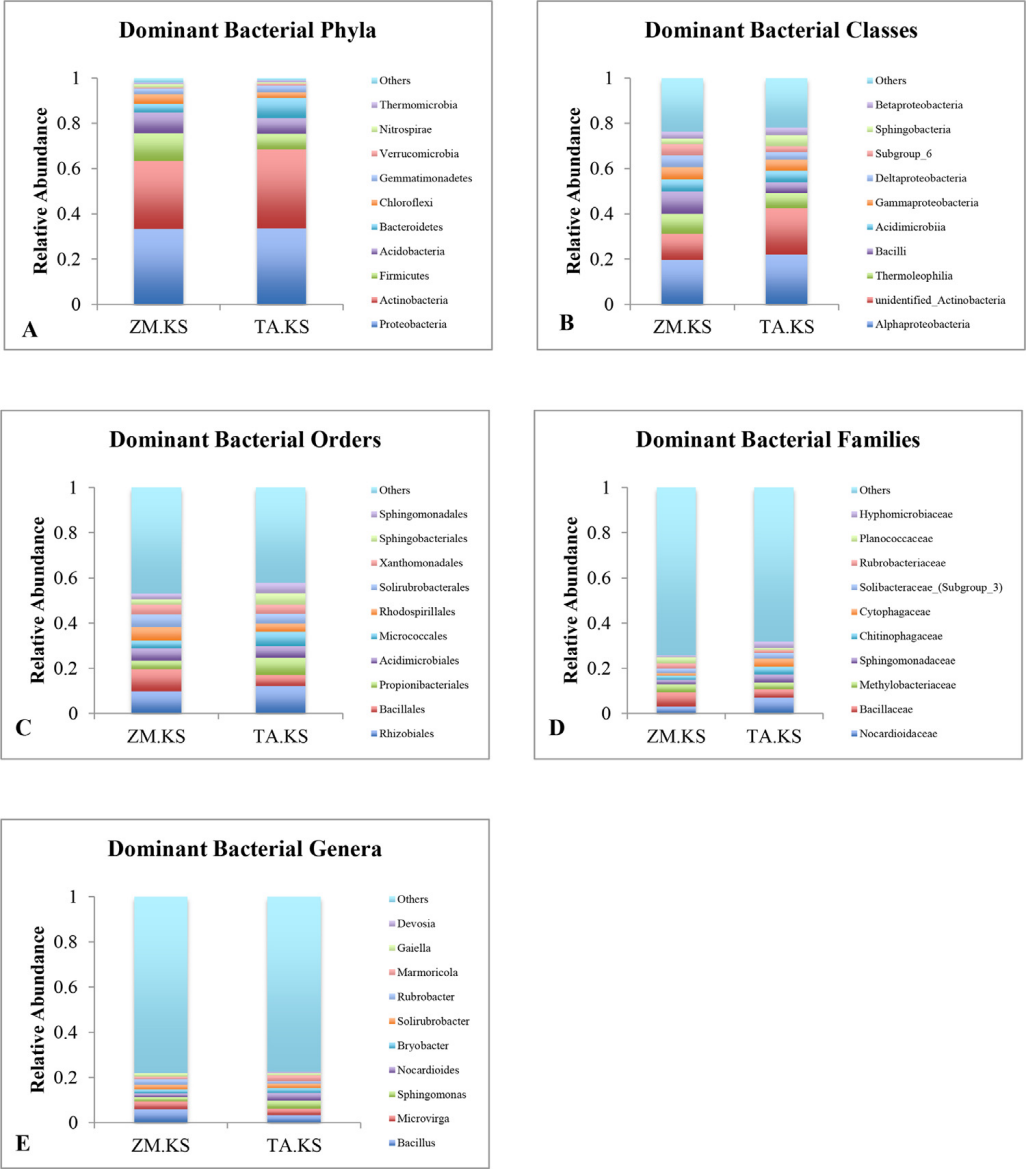
Relative abundance of the dominant bacterial groups residing the rhizosphere of *Zea mays* (ZM.KS) and *Triticum aestivum* (TA.KS).

The dominant bacterial phyla residing rhizosphere of *Zea mays* and *Triticum aestivum* were *Proteobacteria, Actinobacteria, Firmicutes, Acidobacteria, Bacteroidetes, Chloroflexi, Gemmatimonadetes, Verrucomicrobia, Nitrospirae* and *Thermomicrobia*. These phyla accounted for approximately 98 and 98.9% of the bacterial OTUs in *Zea mays* and *Triticum aestivum* respectively (Fig. 2A).

*Dominant bacterial classes.*

*Alphaproteobacteria*, unidentified *Actinobacteria, Thermoleophilia, Bacilli, Acidimicrobiia, Gammaproteobacteria, Deltaproteobacteria, Acidobacteria* Subgroup_6, *Sphingobacteriia* and *Betaproteobacteria* were the dominant bacterial classes accounting approximately 76.3 and 78.2% of the bacterial community in rhizosphere of *Zea mays* and *Triticum aestivum* (Fig. 2B).

*Dominant bacterial orders.*

The dominant bacterial orders in both the rhizosphere soils were *Rhizobiales, Bacillales, Propionibacteriales, Acidimicrobiales, Micrococcales, Rhodospirillales, Solirubrobacterales, Xanthomonadales, Sphingobacteriales* and *Sphingomonadales*. These predominant orders accounted for around 53 and 57.8% of bacterial community in rhizosphere of *Zea mays* and *Triticum aestivum* respectively (Fig. 2C).

*Dominant bacterial families.*

The prominent bacterial families present in the rhizosphere of *Zea mays* and *Triticum aestivum* were *Nocardioidaceae, Bacillaceae, Methylobacteriaceae, Sphingomonadaceae, Chitinophagaceae, Cytophagaceae, Solibacteraceae*_(Subgroup_3), *Rubrobacteriaceae, Planococcaceae* and *Hyphomicrobiaceae* (Fig. 2D). The ten dominant bacterial families formed 25.7% and 31.7% of the bacterial community in rhizosphere of *Zea mays* and *Triticum aestivum,* respectively.

*Dominant bacterial genera.*

The major bacterial genera found in the rhizosphere of *Zea mays* and *Triticum aestivum* were *Bacillus, Microvirga, Sphingomonas, Nocardioides, Bryobacter, Solirubrobacter, Rubrobacter, Marmoricola, Gaiella* and *Devosia* (Fig. 2E). The ten predominant genera accounted for 22% and 22.6% of the bacterial community in the rhizosphere of *Zea mays* and *Triticum aestivum*.

#### 1.2.5 Alpha diversity

Alpha diversity was measured using observed species, chao1, abundance based coverage estimator (ACE), shannon, shannon-pielou, simpson and diversity inverse simpson. The observed species, chao1 and ACE indices reflected species richness. Whereas Shannon, Shannon-pielou, simpson and diversity inverse simpson indices depicted species diversity, accounting both species richness and species evenness. The results showed the presence of great diversity of bacteria in rhizosphere of *Zea mays* and *Triticum aestivum*. Slight variations were observed in the alpha diversity indices of bacterial communities of *Zea mays* and *Triticum aestivum* rhizosphere (Table 2).

**Table 2:** Alpha diversity of bacterial community inhabiting the rhizosphere of *Zea mays* (ZM.KS) and *Triticum aestivum* (TA.KS) at grain filling stage.

Sample	Observed Species	CHAO1	Abundance Based Coverage Estimator	Shannon	Shannon-Pielou	Simpson	Diversity Inverse Simpson
ZM.KS	1340	1440	1418	6.199	0.861	0.995	211.853
TA.KS	1327	1397	1389	6.197	0.862	0.996	236.405

#### 1.2.6 Comparison of the rhizospheric microbiome profiles between two crops

**Fig. 3:**
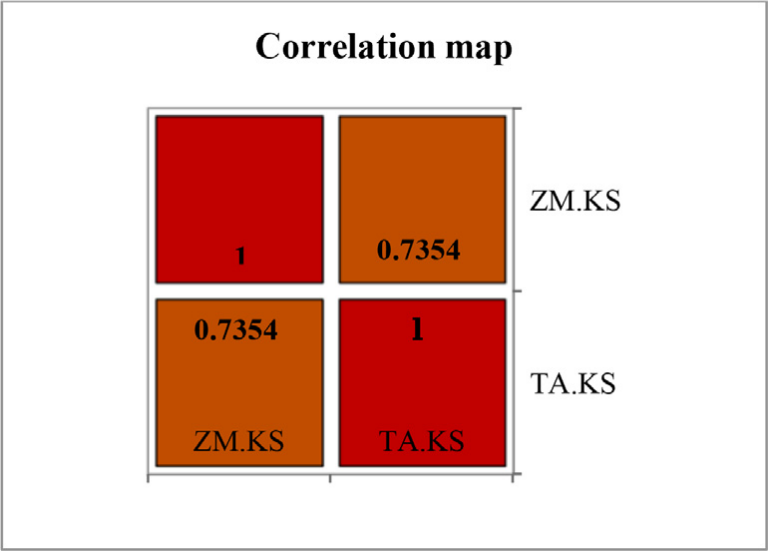
Correlation map depicting similarity between the rhizosphere bacterial community of *Zea mays* (ZM.KS) and *Triticum aestivum* (TA.KS). Correlation coefficients can vary between −1 to 1. Correlation map uses blue red scale, where blue color corresponds to −1 and red color corresponds to 1.

**Fig. 4:**
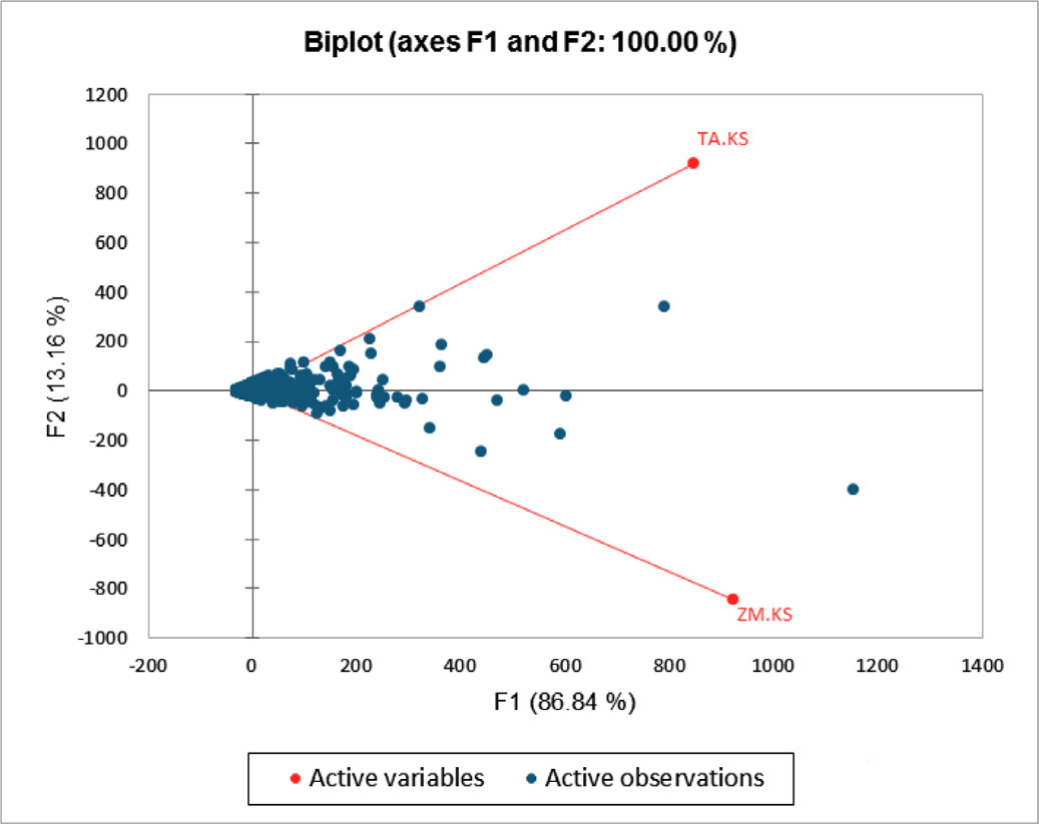
Principal component analysis to reveal variation between the rhizosphere bacterial community of *Zea mays* (ZM.KS) and *Triticum aestivum* (TA.KS). The angle between the vectors represents the relation between variables; Greater the angle between two vectors greater is the variation.

Correlation test revealed positive correlation between *Zea mays* and *Triticum aestivum* bacterial communities (Fig. 3). Principal Component Analysis depicted variation in the core bacterial communities colonizing the rhizosphere of *Zea mays* and *Triticum aestivum* (Fig. 4).

**Fig. 5:**
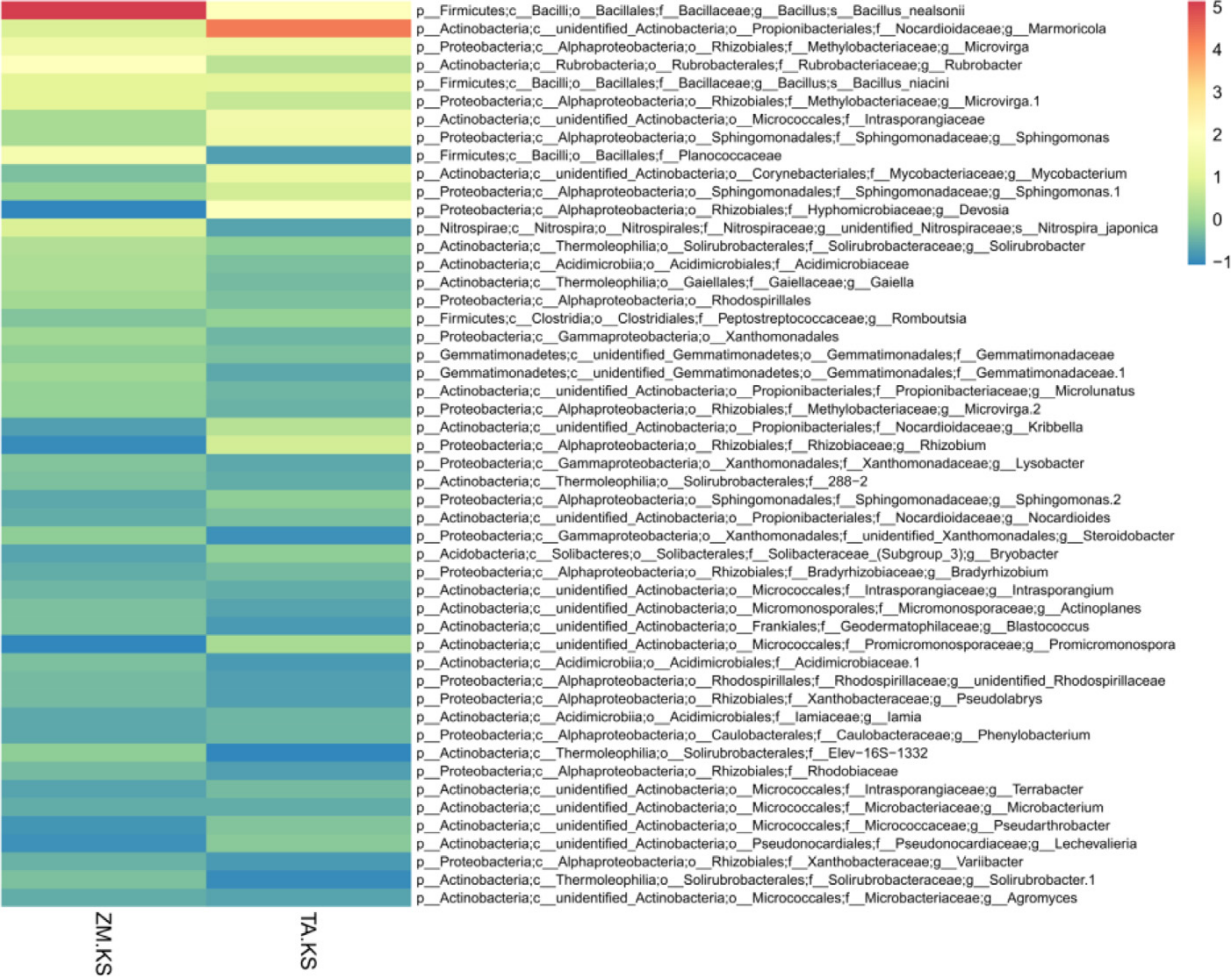
Heatmap illustrating variations in the relative abundance of top 50 bacterial OTUs in the rhizosphere of *Zea mays* (ZM.KS) and *Triticum aestivum* (TA.KS).

Most of the variation between the rhizospheric bacterial communities was attributed to *Bacillus nealsonii* (OTU_01), *Marmoricola* species (OTU_5), *Devosia* species (OTU_32), *Planococcaceae* species (OTU_10), *Rubrobacter* species (OTU_12), *Mycobacterium* species (OTU_25) and *Microvirga* species (OTU_6). Heatmaps depicted the variations in the relative abundance of top 50 OTUs of bacteria (Fig. 5).

## 2 Experimental Design, Materials and Methods

### 2.1 Site description and sample collection

The bulk soil and rhizosphere samples were collected from Kallar Syedan, Pakistan located between 33.42 N° latitude and 73.37 E° longitude. Kallar Syedan has an average annual temperature of 22.1 °C and receives an average annual precipitation of 859 mm.

For soil analysis, 500 g of bulk soil was collected independently from 1 to 6 inches and 7 to 12 inches depths. The bulk soil samples were sent to the Soil and Water Testing Laboratory, Rawalpindi, Pakistan for analysis. The parameters analyzed were electrical conductivity, pH, organic matter, available phosphorus, potassium, saturation percentage and texture of soil.

*Triticum aestivum* and *Zea mays* rhizosphere samples were collected in April and September 2018, respectively. For sample collection, soil was dug to 10 cm and plant along with soil was taken and packed in a resealable bag. Six replicates of both *Triticum aestivum* and *Zea mays* were excised from different locations of same field at the grain filling stage. Samples were sent to Microbiology Laboratory at Allama Iqbal Open University, Islamabad, Pakistan within few hours and stored at −80 °C.

### 2.2 Rhizosphere sample preparation and DNA isolation

The loosely attached soil was removed by shaking the roots. The remaining attached soil was collected and sieved to remove tiny root leftovers. The cleaned rhizosphere soil was grinded to fine powder and used for DNA isolation. High quality microbial DNA was extracted using Invitrogen™ Pure Link™ Microbiome DNA Purification kit (ThermoFisher Scientific, Catalog No. A29790, Pub. No. MAN0014331). The DNA was isolated from 200 mg of the rhizosphere soil using manufacturer's protocol with some modifications. The quality and purity of DNA was monitored on 1% agarose gel. The DNA isolated from biological replicates was pooled and subjected to PCR amplification.

### 2.3 Amplicon generation

The V3-V4 region of 16S rRNA gene was amplified using universal primers (341F and 806R) with the barcode. The Phusion® High-Fidelity PCR Master Mix (New England Biolabs) was used for PCR reactions. The Phusion® High-Fidelity PCR Master Mix manual was followed for preparation of reaction cocktail and PCR profile. Gel electrophoresis was performed using 2% agarose gel. The products between 400 and 450 bp were chosen for purification. The PCR products were purified through Qiagen Gel Extraction Kit (Qiagen, Germany) following manufacturer's protocol.

### 2.4 Library preparation and sequencing

To generate sequencing libraries, NEB Next ® Ultra DNA Library Prep Kit for Illumina was used following manufacturer's instruction. The quality of the libraries was accessed via Qubit @ 2.0 Fluorometer and Agilent Bioanalyzer 2100 system. Finally, the libraries generated were sequenced using IonS5™XL (Thermofisher) platform.

### 2.5 Data analysis

#### 2.5.1 Single-end reads, data filtration and chimera removal

Each sample was assigned single-end reads on the basis of its unique barcode. The single-end reads were truncated by cutting off primer sequence and barcode. The raw reads were filtered to obtain high-quality clean reads under specified filtering conditions using Quantitative Insights Into Microbial Ecology software (Qiime Version 1.7.0) (http://qiime.org/scripts/ split_libraries_fastq.html) [1]. To obtain the effective tags, the clean reads were compared with reference Gold database (http://drive5.com/uchime/uchime_download.html) for detection and removal of chimera sequences using UCHIME algorithm (http://www.drive5.com/usearch/ manual/uchime_algo.html) [2].

#### 2.5.2 OTU cluster and species annotation

All the effective reads were subjected to sequence analysis using Uparse software (Version 7.0.1001) (http://drive5.com/uparse/) [3]. The sequences depicting ≥ 97% similarity were assigned to same operational taxonomic units (OTUs). The representative sequence of each OTU was selected for further annotation. For representative sequences of 16S rRNA, the SSUrRNA database of SILIVA Database (http://www.arb-silva.de/) [4] was used for annotation at each taxonomic rank using Mothur software with threshold 0.8∼1 [5]. The Korona figures were constructed using maximum depth to visually display species annotation results [6].

### 2.6 Alpha diversity

Alpha diversity was used to determine the complexity of species diversity within a sample. Alpha diversity was measured via alpha diversity indices that included observed species, chao1, abundance based coverage estimator, shannon, Shannon-pielou, simpson and diversity inverse simpson. OTUs abundance information was normalized using a standard of sequence number corresponding to the sample with the least sequences. The R-software (version 3.6.3) was used to determine the alpha diversity for each sample using the normalized data. The packages used to calculate the alpha diversity indices were phyloseq, microbiome, knitr and dplyr.

### 2.7 Comparison of the rhizospheric microbiome profiles between two crops

Correlation test and Principal Component Analysis were performed to reflect the correlation and variation between the microbial community composition inhabiting the rhizosphere of *Zea mays* and *Triticum aestivum*. Correlation test and Principal Component Analysis were done by XLSTAT-2021 using normalized data to compare the rhizosphere microbiome of *Triticum aestivum* and *Zea mays*. Heatmap for the top 50 OTUs was obtained via Clustalvis.

## CRediT authorship contribution statement

**Sadia Latif:** Conceptualization, Methodology, Data curation, Software, Writing – original draft. **Rizwana Kousar:** Conceptualization, Software, Writing – original draft. **Anum Fatima:** Methodology, Data curation. **Hina Fatimah:** Conceptualization, Writing – review & editing. **Saba Farooq:** Software, Validation. **Naeem Khan:** Writing – review & editing. **Tayyaba Andleeb:** Writing – original draft. **Tariq Shah:** Writing – review & editing.

## Declaration of Competing Interest

The authors declare that they have no known competing financial interests or personal relationships that could have appeared to influence the work reported in this paper.

## Footnotes

Supplementary material associated with this article can be found in the online version at doi:10.1016/j.dib.2022.108057.

## References

[1] Bokulich N.A., Subramanian S., Faith J.J., Gevers D., Gordon J.I., Knight R., Mills D.A., Caporaso J.G. (2013). Quality-filtering vastly improves diversity estimates from Illumina amplicon sequencing. Nat. Methods.

[2] Edgar R.C., Haas B.J., Clemente J.C., Quince C., Knight R. (2011). UCHIME improves sensitivity and speed of chimera detection. Bioinformatics.

[3] Edgar R.C. (2013). UPARSE: highly accurate OTU sequences from microbial amplicon reads. Nat. Methods.

[4] Wang Q., Garrity G.M., Tiedje J.M., Cole J.R. (2007). Naive Bayesian classifier for rapid assignment of rRNA sequences into the new bacterial taxonomy. Appl. Environ. Microbiol..

[5] Quast C., Pruesse E., Yilmaz P., Gerken J., Schweer T., Yarza P., Peplies J., Glöckner F.O. (2012). The SILVA ribosomal RNA gene database project: improved data processing and web-based tools. Nucleic. Acids. Res..

[6] Ondov B.D., Bergman N.H., Phillippy A.M. (2011). Interactive metagenomic visualization in a Web browser. BMC Bioinf..

